# The role of aberrant promoter hypermethylation of DACT1 in bladder urothelial carcinoma

**DOI:** 10.7555/JBR.26.20110099

**Published:** 2011-04-12

**Authors:** Huan Cheng, Zhonglei Deng, Zengjun Wang, Wei Zhang, Jiantang Su

**Affiliations:** Department of Urology, the First Affiliated Hospital of Nanjing Medical University, Nanjing, Jiangsu 210029, China.

**Keywords:** DNA methylation, DACT1, hypermethylation, bladder urothelial carcinoma

## Abstract

The purpose of this study was to determine the relationship between hypermethylation of *DACT1* gene promoter and lower mRNA expression in bladder urothelial carcinoma tissue. The methylation status of 29 urothelial carcinoma samples and 29 normal tissue samples were examined by methylation-specific polymerase chain reaction (MSP). The *DACT1* mRNA transcript levels and DACT1 protein levels in all samples were then evaluated to define the relationship between the methylation status of the *DACT1* promoter and its expression at the transcriptional and translational levels. Decreased expression of DACT1 was detected in 89.66% of urothelial carcinomas (26/29; *P* < 0.005). Promoter hypermethylation was found in 58.62% (17/29) urothelial carcinomas and 25% (7/29) normal tissues, respectively (*P* < 0.05). DACT1 expression was lower in tissues where the *DACT1* gene promoter was hypermethylated than in unmethylated tissues (0.25±0.17 *vs* 0.69±0.30, *P* < 0.05). *DACT1* gene hypermethylation was closely related to tumor size, grade and stage (*P* < 0.05). Our results indicate that silencing and downregulation of *DACT1* mRNA may be implicated in carcinogenesis and the progression of bladder urothelial carcinoma, and may be a potential prognostic factor.

## INTRODUCTION

Bladder cancer is the sixth most common cancer in the world[1]. The majority of bladder cancers are comprised of urothelial carcinoma, also known as transitional cell carcinoma. Although the oncogenesis of bladder cancer is unclear so far, it is generally agreed that the accumulation of multiple genetic and epigenetic alternations leading to the activation of proto-oncogenes and/or inactivation of tumor-suppressor genes contribute to the development of bladder cancer[2]–[4].

Aberrant DNA methylation is now considered to be the most important epigenetic alteration in many cancers[5], including bladder cancer[6]. In general, DNA methylation is one of the best-studied epigenetic alterations in human cancers and may play important roles in carcinogenesis[7]. Carcinogenesis is associated with changes in this epigenetic phenomenon, including two distinct and seemingly opposing trends: global decrease in cytosine methylation (hypomethylation) and methylation of cytocine in CpG islands (hypermethylation)[8]. Such alterations can result from DNA mutations or deletions, or from epigenetic alterations, e.g., changes in gene expression that are not mediated by a change in the nucleotide sequence, such as DNA promoter hypermethylation[9]. Hypermethylation of normally unmethylated tumor suppressor genes correlates with a loss of expression in cancer cell lines and primary tumors, suggesting that hypermethylation of tumor suppressor genes could promote carcinogenesis.

The *DACT1* gene was initially found as a signal regulation molecule in the clawed frog[10]. It is the principal member of the Dact family. The *DACT* gene is well conserved at the genomic level during evolution. The *DACT1* gene maps to chromosome 14, at 14q23.1. Many studies have demonstrated that DACT1 is a key mediator in the negative regulation of the Wnt/β-catenin signaling pathway, whereas abnormal activation of this signaling pathway participates in the process of cancer progression[11]-[13]. A growing body of evidence has emerged in the past decade on the involvement of the *DACT* gene in the pathogenesis of cancer. Several studies have reported aberrant expression of a number of *DACT* genes in cancers. The examples include *DACT1* in stomach neoplasms, liver cancer and germ cell tumor, and *DACT2* in endometrial adenocarcinoma[14]. However, up to now, the role of DACT1 in bladder urothelial carcinoma has not yet been reported and whether DNA methylation participates in bladder carcinogenesis is also unclear.

In this study, the methylation status of the *DACT1* gene was examined in tissue from 29 normal bladders and tissue from 29 bladder urothelial carcinomas. We also examined the expression level of *DACT1* mRNA and protein in all samples. Using methylation-specific polymerase chain reaction (MSP) and reverse transcription polymerase chain reaction (RT-PCR), a clear relationship was found between hypermethylation status and the low expression of *DACT1* in bladder transitional cell carcinomas. *DACT1* was hypermethylated in almost all human bladder cancers but unmethylated in normal tissues. We hypothesized that these changes could be of diagnostic value for the detection of bladder cancer and decided to measure the degree of DNA methylation in *DACT1* in bladder transitional cell carcinoma tissues.

## MATERIALS AND METHODS

### Tissue samples

We obtained frozen tissue samples of 29 bladder carcinomas and 29 normal bladder tissues from the First Affiliated Hospital of Nanjing Medical University. The study was approved by the Institutional Human Investigations Committee. All patients signed the informed consent. The tumors were verified as transitional cell carcinomas by two experienced pathologists according to the WHO criteria[15]. The patients had well-documented clinical history and follow-up information.

### Isolation of genomic DNA

Bladder cancer tissues and normal bladder tissues were obtained after surgical resection and snap-frozen and stored at -70°C. Genomic DNA was isolated by using a Promega Wizard DNA isolation kit. The tissues were incubated at 55°C in a homogenization buffer containing 50 mmol/L Tris (pH 8.0), 1 mmol/L EDTA, 0.5% Tween 20, and 5 mg/mL proteinase K for 3 h prior to genomic DNA isolation.

### RT-PCR for *DACT1*

Total RNA was extracted with Trizol reagent (Invitrogen, Carlsbad, CA, USA), and cDNA was synthesized from total RNA with oligo-dT primer (Reverse Transcription System, Promega, Madison, WI, USA) following the manufacturer's instructions. Primer sequences for *DACT1* amplification were 5′-GCGAAGAGATGCTGGTITGT-3′ (sense) and 5′-AAATTGTGGTGTGGAGAGGG-3′ (antisense). The PCR included 2 min of denaturation at 95°C followed by 35 cycles of 30 s at 94°C, 30 s at 55°C and 30 s at 72°C, and a final extension for 7 min at 72°C. Primer sequences for *β-actin* amplification were 5′-GTGGGGCGCCCCAGGCACCA-3′ (sense); antisense: 5′-CTCCTTAATGTCACGCACGATTTC-3′ (antisense). A BLAST search confirmed that these primer sequences were identical to the endogenous human target genes. The PCR products were resolved by electrophoresis in a 1.5% agarose gel and stained with ethidium bromide.

### DNA extraction and methylation-specific PCR

Genomic DNA was obtained by digestion with proteinase K (Sigma, St. Louis, MO, USA), followed by phenol/chloroform (1:1) extraction. Briefly, 4 ng genomic DNA was denatured by NaOH and modified by sodium bisulfite. The DNA was purified using a Wizard DNA purification kit (Promega), desulfonated with NaOH, precipitated with ethanol, and resuspended in water. PCR was performed with bisulfite-treated DNA as a template using specific primer sequence for methylated and unmethylated forms of the genes. The primer sequences of *DACT1* for methylated reaction were as follows: 5′-ACTACTAATCAAAAACGCCCTACG-3′; (sense) and 5′-AATAGTCGTGTTTTATITTCGGGTAC-3′ (antisense). The sequences for the unmethylated reaction were 5′-AAAACTACTAATCAAAAACACCCTACAC-3′ (sense) and 5′-AT AGTTGTGTTTTATTTTTGGGTATGA-3′ (antisense).

Step-down PCR was performed in 20-µL reaction buffer containing 10×loading buffer (including Mg^2+^), 2 µL dNTP, 1 µL each PCR primer, 0.2 µL AmpliTaq polymerase, 2 µL bisulfite-modified DNA, and 16.8 µL diethyl-pyrocarbonate-treated water. The reaction was started by heating the samples at 95°C for 12 min followed by 35 cycles at 94°C for 30 s, 55°C (methylated) or 53°C (unmethylated) for 30 s, and 72°C for 30 s, followed by a final extension at 72°C for 7 min. The amplification products were separated on a 1.5% agarose gel and visualized by UV illumination. The results were confirmed by repeating bisulfite treatment and methylation-specific PCR for all samples.

### Real-time PCR

Real-time quantitative PCR analysis was performed on cDNAs, using the ABI7300 instrument. PCR samples were prepared in a final volume of 20 µL containing TagHS (5 U/µL), 10× buffer (Mg^2+^ plus), dNTP mixture (2.5 µmol/L), forward primer (20 µmol/L), reverse primer (20 µmol/L), 2 µL cDNA template, and 20× Eva Green (1 µL). PCR primer sets were as follows: *β-actin*: sense 5′-GTGGGGCGCCCCAGGCACCA-3′ and antisense: 5′-CTCCTTAATGTCACGCACGATTTC-3′; *DACT1*: sense:5′-GCGAAGAGATGCTGGTITGT-3′ and antisense: 5′-AAATTGTGGTGTGGAGAGGG-3′. The PCR was run at 50°C for 2 min, 95°C for 10 min, 95°C for 15 s, and 60°C for 1 min. Melting curve analysis was used to monitor the accumulation of the PCR products. *β-actin* mRNA was used as internal control to standardize the results of different mRNA expression levels. Triplicate Ct values were analyzed in Microsoft Excel using the comparative CT (▵▵CT) method as described by the manufacturer (Applied Biosystems, Foster City, CA, USA). The results were normalized against the *β-actin* control.

### Western blot analysis

Tissue samples were homogenized in ice-cold lysis buffer (50 mmol/L Tris (pH 8.0), 100 mmol/L NaCl, 0.1% SDS, 1% NP-40, 0.5 mmol/L EDTA) containing a complete protease inhibitor cocktail (Roche, Mannheim, Germany). Proteins (20 µg) were boiled for 5 min and separated with 15% SDS-PAGE, and then transferred onto a nitrocellulose membrane (Bio-Rad, Hercules, CA, USA), blocked in 5% skim milk in phosphate buffered saline (PBS), and probed with a goat-anti-human DACT1 antibody (1:2,000 dilution, Santa Cruz Biotechnology, Santa Cruz, CA, USA), followed by incubation with an anti-rabbit IgG secondary antibody.

**Fig. 1:**
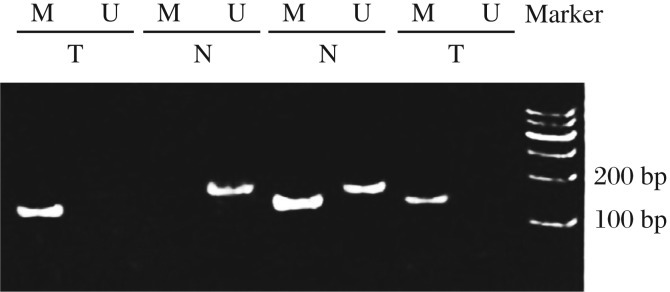
The *DACT1* gene is hypermethylated in bladder cancer tissues. Methylation status of the promoter region CpG island of the *DACT1* gene in bladder cancer and normal tissues was examined by methylation-specific polymerase (MSP) chain reaction. U: unmethylated (183 bp); M: methylated (181 bp). T:tumor tissues; N: normal tissues.

### Statistical analysis

All statistical analyses were performed by SPSS version 13.0 for Windows (SPSS Inc., Chicago, IL, USA). The difference between unmethylated and hypermethylated tissues was assessed by χ^2^ or Fisher's exact test. A Student's *t*-test was used for statistical comparison in RT-PCR analysis. *P* < 0.05 was considered statistically significant.

## RESULTS

### The methylation status of the DACT1 gene

**Fig. 2:**
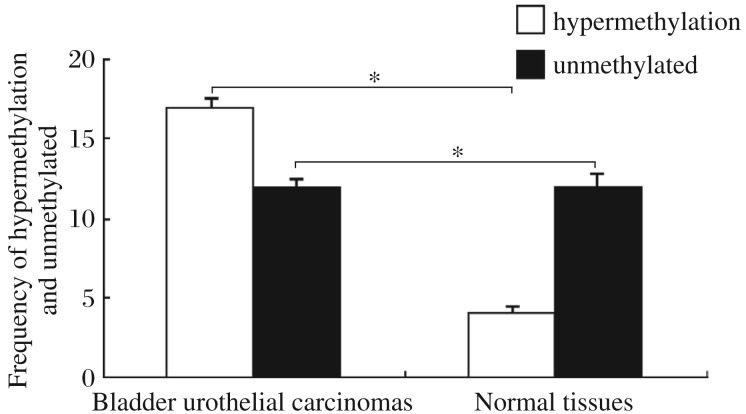
Methylation-specific PCR analysis of the *DACT1* promoter from normal and bladder cancer tissues. The MSP assay showed that the frequency of hypermethylaion was 25% and 58.62% in normal tissue and bladder cancer tissues, respectively. *DACT1* was hypermethylated in most bladder cancer tissues, and unmethylated in normal tissues. *P < 0.05.

The methylation-specific PCR results showed that the *DACT1* gene was mostly methylated in bladder cancer tissues (***Fig. 1***). The promoter of the *DACT1* gene in 17 out of 29 (58.62%) cancer tissue specimens and in 7 out of 29 (25%) normal tissues was hypermethylated (*P* < 0.05) (***Fig. 2***). Thus, these data revealed that *DACT1* hypermethylation may be a neoplastic feature of bladder cancers. The expression of *DACT1* was low in bladder cancer tissues.

**Fig. 3:**
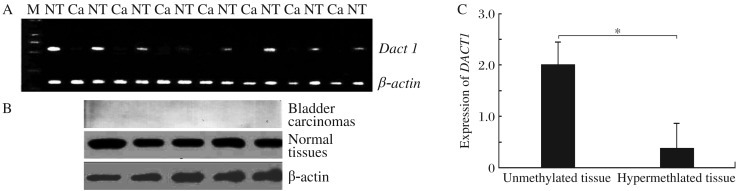
The expression of the DACT1. A: RT-PCR analysis of the *DACT1* expression in 29 bladder cancer samples and 29 normal bladder tissue samples. The expression of the *DACT1* mRNA was low, even absent in most bladder carcinomas, but high in normal tissue. *β-actin* was used as an internal control. Ca: cancer tissue; NT: normal tissue. B: Western blotting analysis of DACT1 expression had a trend similar to the mRNA expression: lower expression in bladder carcinoma samples compared to normal control. DACT1 protein expression is related to hypermethylation status: lower expression is associated with hypermethylation of *DACT1* gene promoter. C: RT-PCR confirms the differential expression of *DACT1* mRNA between the hypermethylated and unmethylated promoter of *DACT1* gene (*β-actin* housekeeping gene as an internal control). *P < 0.05.

### The expressionof the *DACT1*gene

*DACT1* mRNA expression was absent in most bladder carcinomas, but present in almost all normal bladder tissue (***Fig. 3A***). Western blot analysis confirmed that DACT1 protein was absent in bladder cancer tissues. Conversely, DACT1 protein was present in almost all normal samples (***Fig. 3B***). DACT1 protein was detected in some bladder cancers, but at a significantly reduced level in comparison to normal controls. The RT-PCR assay confirmed the quantitative relationship between the hypermethyaltion status and DACT1 expression (***Fig. 3C***): the expression of DACT1 in the hypermethylated group was lower than that in the unmethylated group (0.25±0.17 *vs* 0.69±0.30, *P* < 0.05).

### Correlation between methylation status of the *DACT1* gene and clinicpathological data in bladder carcinomas patients

*DACT1* gene hypermethylation was closely associated with tumor size, grade and stage (*P* < 0.05), but not gender and age (***Table 1***), suggesting that the methylation rate of the *DACT1* gene increased with the progression of bladder cancer.

## DISCUSSION

In the United States, there were approximately 68,810 newly diagnosed bladder cancer cases and 14,100 deaths were expected in 2008[16]. More than 90% of bladder carcinomas have a transitional cell origin[16]. Substantial evidence has demonstrated that many tumor suppressor genes are methylated. Shi *et al*.[17] found that aberrant methylation in the *DBC2* promoter may be responsible for the loss of DBC2 expression in bladder cancer and this hypermethylation event could play a crucial role in the early stage of bladder tumorigenesis. Eissa *et al*.[18] demonstrated that methylated *RARβ2* and *APC* are significantly higher in bladder cancer (62.8%, 59.5%) than benign tumors (16.4%, 5%), and virtually undetectable in healthy volunteers (0%) (*P* < 0.0001). Hypermethylation of tumor suppressor genes *SFRP1* and *SOCS*-*1* correlates with bladder carcinogenesis and development[19]. Methylation of tumor suppressor genes plays an important role in the tumorigenesis of bladder cancer. Substantial evidence has demonstrated that tumor cells display mostly hypermethylation profiles in suppressor genes, which contributes to the suppression of their expression[20].

In the present study, we first examined the hypermethylation status of DACT1 in normal and bladder cancer tissue, as well as the levels of *DACT1* mRNA transcripts and protein. Hypermethylation was detected in bladder carcinomas by methylation specific PCR. The mono-alleles were detected in most bladder cancers. The *DACT1* gene was hypermethylated in 58.62% (17 out of 29) urothelial carcinomas, and only 25% (7 of 29) in healthy bladder tissue (*P* < 0.05). These results indicate that the methylated type, which might occur either randomly or in mono-allelic manner, can be found in bladder cancer and methylation alone could repress DACT1 expression in bladder cancer. Although some cases (including normal tissues and bladder cancers) showed a hypomethylation status (methylated and unmethylated alleles), the *DACT1* expression levels proved different by RT-PCR assays. Based on these findings, we hypothesize that the ratio of unmethylated DACT1 is higher, and the expression level of DACT1 may increase. In other words, the methylation status in some genes determines their expression level in tissues. Western blot analysis showed that DACT1 expression is lower in bladder cancer and the trend was the same as the hypermethylation status of the *DACT1* gene. Additionally, we analyzed the correlation between methylation status of the *DACT1* gene and clinicopathological features. The results suggest that increasing DACT1 methylation is correlated with tumor growth and progression. We also detected methylation in some normal bladder tissue samples, suggesting that the presence of other mechanisms, such as histone modification, may also regulate the expression of DACT1. Together with the fact that methylation of the *DACT1* CpG island is common in bladder cancer, these findings indicate that DACT1 hypermethylation is related to the progression of bladder transitional cell carcinomas. Our data also suggest that other molecular mechanisms contribute to lower DACT1 expression in the tumor.

**Table 1:** Correlation between methylation status of the *DACT1* gene and clinicopathological characteristics in bladder urothelial carcinoma patients.

Variables	Cases (*n*)	Total methylation rate (%)
Sex		
Male	15	20.6
Female	14	21.3
*P* value		0.936
Age (year)		
≤60	26	34.6
> 60	3	35.3
*P* value		0.945
Tumor size (cm)		
< 4	16	18.1
≥4	13	81.8
*P* value		0.016
T stage		
T1/2	15	13.7
T3/4	14	77.4
*P* value		0.021
N stage		
N0	18	26.2
N1	11	56.4
*P* value		0.029
M stage		
M0	20	24.6
M1	9	66.4
*P* value		0.032
Grade		
0	2	0
I	8	12.4
II	7	23.2
III	9	56.3
IV	3	66.6
*P* value		0.038

Data were analyzed with Fisher's exact test.

Paradoxical aberration of DNA methylation pattern is a hallmark of cancer. With a global loss of DNA methylation that coexists with regional hypermethylation of certain genes[21], it has been proposed that hypermethylation and hypomethylation in cancer are two independent processes and target different programs at different stages in tumorigenesis[22]. Hypermethylation and silencing of genes regulating proliferation are proposed to be critical for deregulating growth in early carcinogenesis, whereas hypomethylation and activation of other genes may be more important for metastasis[23]–[25]. The importance of hypermethylation and inactivation of tumor suppressor genes is well-documented in various cancers, but the role of DNA hypomethylation in advanced cancers and metastasis has been less thoroughly studied and remains hypothetical[26].

In conclusion, our study showed that hypermthylation could regulate DACT1 expression in bladder transitional cell carcinomas. From a clinical point of view, DACT1 could potentially become a target for intervention. Better understanding of the mechanism of DACT1 expression may eventually lead to novel treatment for bladder transitional cell carcinomas.
